# A Case Report of Acute Appendicitis Complicated by Appendicoliths

**DOI:** 10.5070/M5.52213

**Published:** 2026-04-30

**Authors:** Faris F Halaseh, Lindsey C Spiegelman

**Affiliations:** * University of California, Irvine, School of Medicine, Irvine, CA; ^ University of California, Irvine, Department of Emergency Medicine, Orange, CA

## Abstract

This case report highlights the clinical complexities of diagnosing and managing appendicitis complicated by appendicoliths in a 44-year-old female patient. Appendicitis is a common cause of acute abdominal pain, with imaging playing a crucial role in diagnosis. The patient presented with symptoms of right lower quadrant pain, nausea, vomiting, and chills that were initially misattributed to menstrual cramps. Diagnostic evaluation revealed multiple appendicoliths via computed tomography (CT), and the decision for laparoscopic appendectomy was based on the imaging results and clinical presentation. Intraoperative findings showed gangrenous but non-perforated appendicitis. The patient’s postoperative recovery was uneventful, with successful symptom resolution. This case highlights the importance of considering appendicitis in the differential diagnoses for acute abdominal pain in females, particularly with overlapping gynecological symptoms. The high sensitivity of CT imaging for appendicitis and the necessity of timely surgical intervention in cases involving appendicoliths are discussed. Multimodal imaging and prompt surgical management are vital for favorable outcomes in complicated appendicitis cases.

**Topics:**

Appendicolith, CT scan, right lower quadrant pain, abdomen, gastrointestinal.

**Figure:**
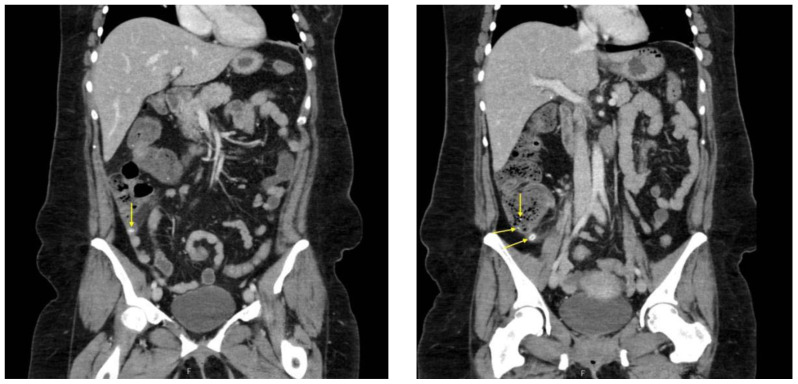
Video Link: https://youtu.be/9tStgnEHqZw

## Brief introduction

Appendicitis is a frequent cause of acute abdominal pain in both adults and children, with males facing a lifetime risk of 8.6% and females a risk of 6.7%.^1^ Because the appendix is a blind-ending tubular loop of bowel, it is prone to collecting particulates from the cecum and ascending colon.^2^ The aggregation of trapped fecal particulates, mineral deposits, and mucus builds up within the lumen of the appendix over time, leading to calcification and the formation of an appendicolith.^2^,^3^ Once an appendicolith reaches a critical diameter, it obstructs the appendiceal lumen, causing luminal stasis, increased intraluminal pressure, and eventually vascular thrombosis, transmural necrosis, and perforation.^3^

Whether or not caused by an appendicolith, the most common symptom of appendicitis is the acute onset of central abdominal pain migrating to the right iliac fossa. Treatment typically requires emergency surgery to prevent the risk of perforation, which can lead to peritonitis and sepsis.^4^ Most appendiceal fecaliths and calculi are found in the pediatric and young adult age groups; few are found after the age of 35.^5^ Appendicoliths are also identified more frequently in men than in women.^6^ It has been reported that appendicoliths account for approximately 10% of appendicitis cases.^4^

In this report, we describe the case of a 44-year-old female with an appendicolith visible on CT. We discuss the clinical findings, diagnostic workup, treatment, and provide images to improve visualization of the diagnosis. Patient-written consent for publication was obtained during admission.

## Presenting concerns and clinical findings

The patient is a 44-year-old female with a history of cholecystectomy, migraines, fibroids, and tubal ligation who presented to the ED with a one-day history of right lower quadrant (RLQ) pain, nausea, vomiting, and chills. The patient first noticed RLQ abdominal pain one day prior, initially attributing it to severe menstrual cramps. However, the pain persisted and had intensified over the course of 24 hours. The pain was rated as severe and differed in quality from her usual menstrual cramp pain. The RLQ pain radiated to her right lower back, and she had never experienced pain like this before. She was menstruating at presentation, which had lasted for 10 days, and she reported experiencing heavy bleeding. The patient also reported nausea, vomiting, and chills but denied chest pain, shortness of breath, diarrhea, constipation, fever, dysuria, and hematuria.

## Significant findings

On initial evaluation, the patient was afebrile, tachycardic to the 100s, and her bedside hemoglobin was 10.7 g/dL. The remainder of her vital signs were within normal limits. The abdominal examination revealed a soft, non-distended abdomen with tenderness to palpation in the suprapubic region, right upper quadrant, and most notably the right lower quadrant. The patient also exhibited rebound tenderness but no involuntary guarding or costovertebral angle tenderness.

Pertinent laboratory results revealed a white blood cell count (WBC) of 16.0 K/mm.^3^ An electrocardiogram demonstrated a normal sinus rhythm. Urine analysis was positive for hematuria.

Contrast-enhanced CT of the abdomen and pelvis was obtained. Coronal CT demonstrated a dilated appendix measuring up to 1.2 cm in diameter with multiple appendicoliths (yellow arrows), the largest measuring 1.1 cm. Mild periappendiceal fat stranding was present, consistent with acute appendicitis complicated by appendicoliths. The surrounding bowel and colon were normal in caliber and distribution. An additional coronal view demonstrated a dilated appendix (yellow arrow) containing an appendicolith with periappendiceal inflammatory changes. No evidence of perforation, abscess, or drainable fluid collection was identified.

## Patient course

At initial presentation, the differential diagnoses were broad and included acute appendicitis, ovarian cyst rupture, ovarian torsion, colitis, and menstrual cramping. The bedside examination revealed classic signs of appendicitis such as right lower quadrant tenderness and rebound tenderness, while laboratory results showed an elevated white blood cell count, indicating an inflammatory process. Computed tomography imaging confirmed the diagnosis, revealing hallmark features of appendicitis, including multiple appendicoliths and a dilated appendix with periappendiceal fat stranding.

The case was discussed with the emergency general surgery team, and the patient was admitted for a proposed laparoscopic appendectomy. During the operation, the cecum was evaluated, and the appendix was visualized consistent with acute gangrenous appendicitis without frank perforation. The appendix was removed, and the remainder of the operation was uneventful. At the time of discharge, the patient was tolerating a normal diet, ambulating, voiding, and her pain was controlled with oral pain meds. Vital signs remained stable, and she was afebrile. On follow up, the patient was tolerating a solid diet with normal bowel function. There were no signs of infection at incision sites.

## Discussion

Acute appendicitis is among the most frequent acute abdominal emergencies, with an estimated 250,000 yearly appendectomies being performed in the United States alone.^7^ Over the years, advancements in diagnostic and treatment methods have emerged, such as prediction scores, ultrasonographic and CT imaging, laparoscopic surgery, and nonoperative treatments for uncomplicated cases using antibiotic therapy alone.^1^,^8^–^14^

The presence of an appendicolith in cases of appendicitis is clinically significant due to its association with increased complication rates. Patients with appendicoliths have a higher likelihood of experiencing perforation, abscess formation, and post-operative complications compared to those with appendicitis without appendicoliths.^15^,^16^ They are also more likely to cause failure of treatment with antibiotics alone.^17^ Given these risks, timely and accurate diagnosis is crucial.

Although histology and surgery are the gold standards for confirming appendicitis and visualizing appendicoliths, imaging plays a crucial role in diagnosis and preventing unnecessary surgeries. The most commonly used imaging modalities for appendicoliths are plain X-ray, ultrasound (US), and CT.^18^ X-ray diagnoses appendicoliths in only about 10% of patients with appendicitis, making CT the most utilized imaging method. Multidetector CT has been shown to have a sensitivity of 98.5% and a specificity of 98% for diagnosing appendicitis.^19^ The sensitivity and specificity of CT for detecting appendicoliths are 65% and 86%, respectively, with a positive predictive value of 74% for acute appendicitis.^20^ While US is not as sensitive and specific as CT, its lack of ionizing radiation makes it particularly important in diagnosing pediatric patients and pregnant patients.^21^

The decision to proceed with a laparoscopic appendectomy was based on the imaging findings, clinical presentation, and risk of complications if surgery were not to be performed. Laparoscopic appendectomy is the gold standard treatment for acute appendicitis and offers several benefits, including reduced postoperative pain, shorter hospital stays, and faster recovery times.^22^,^23^ Intraoperatively, the appendix was found to be gangrenous but not perforated, consistent with preoperative imaging findings. The patient’s postoperative course was uneventful, highlighting the effectiveness of timely surgical intervention.

This case report emphasizes the importance of considering appendicitis in the differential diagnosis of acute abdominal pain in adult females, especially when typical gynecological conditions are present. The high sensitivity of CT imaging in identifying appendicoliths and the utility of combining it with ultrasound for a thorough evaluation are key takeaways. Prompt surgical intervention, guided by accurate imaging and clinical assessment, can lead to favorable outcomes even in complicated cases.

## Supplementary Information






