# Supplementary figures and images for: A Case Report of Acute Appendicitis Complicated by Appendicoliths
Source: J Educ Teach Emerg Med. 2026 Apr 30;11(2):V1–5. doi: 10.5070/M5.52213 (PMC13152369; doi:10.5070/M5.52213)

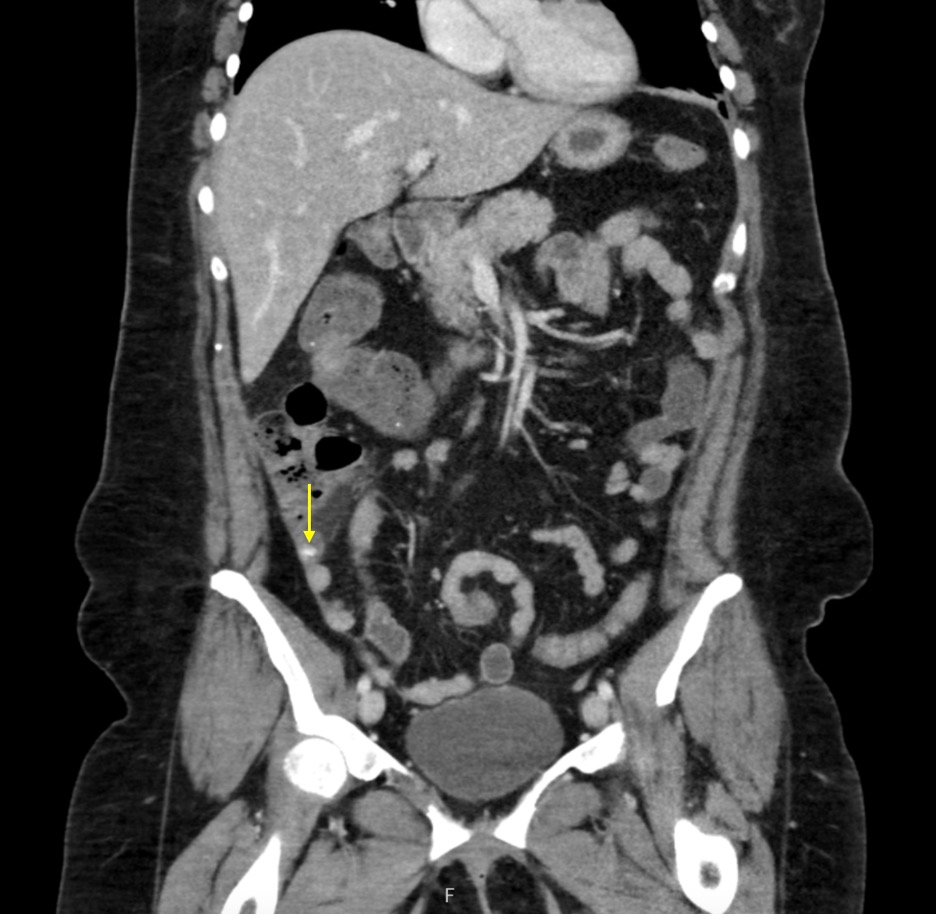

Supplement: Supplementary file 1 [file 11-2-V1-Supp1.jpg]

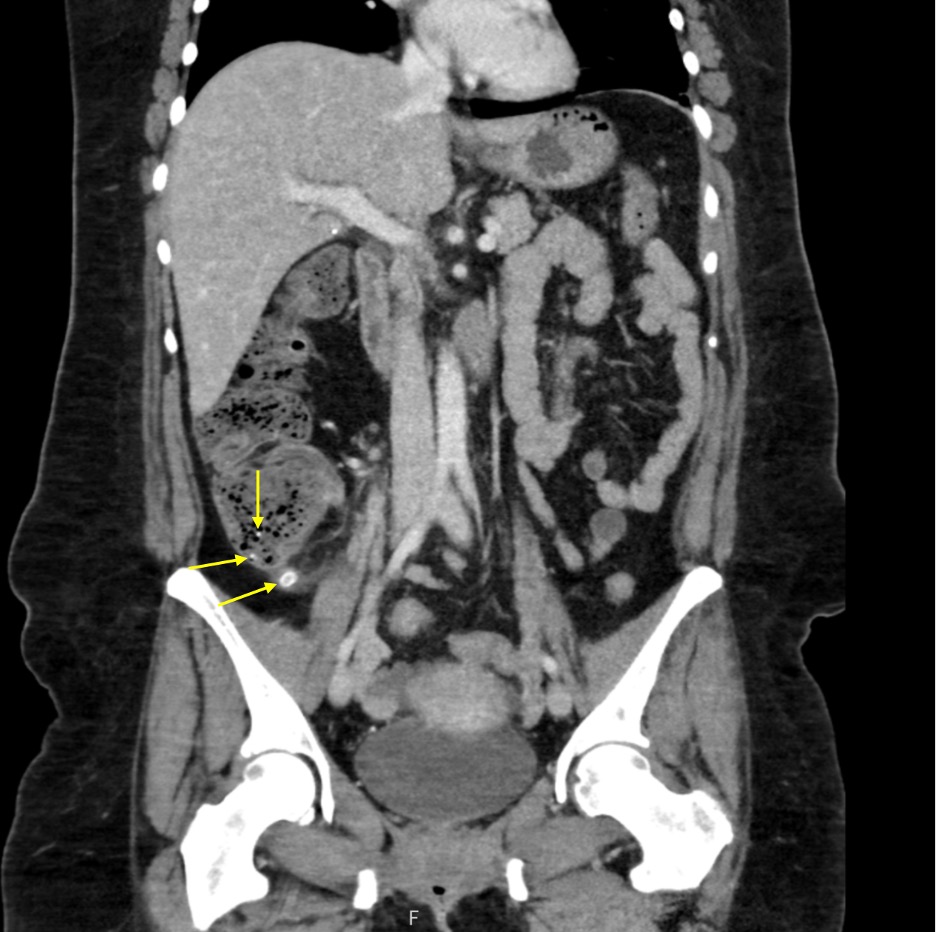

Supplement: Supplementary file 2 [file 11-2-V1-Supp2.jpg]
